# Damiana as a Nerve Tonic

**Published:** 1879-02

**Authors:** 


### EXCERPTA.

Damiana as a Nerve Tonic.—My views on damiana as a sexual tonic are known to a very large number of the members of the medical profession. Further experience has strengthened the high appreciation I have expressed of its value in sexuel debility, and given me, I think, some new ideas as to its physiological action and position as a remedial agent. It is pre-eminently a nerve tonic, impressing the brain and nerve centers very much in the same manner that strychnia does. While, however, void of poisonous properties, it excites nerve cell nutrition, and enables the nerve cell to assimilate its proper pabulum from the blood.

For the medulla oblongata and the medulla spinalis, it has an especial affinity. The motor nerves seem more impressed by its influence than are those of sensation. Hence I inferred that it would prove valuable in paralysis. Opportunities offering, I tested the accuracy of this inference in two cases—one hemiplegic, the other paraplegic. In both, damiana proved of unquestionable efficacy; the advantage was as unequivocal as I ever witnessed from the use of strychnia and ergot.

If my theory of its *modus operandi*—that it acted as an invigorator of the primordial nerve cell—be correct, it is easy to understand its true place in the treatment of certain forms of paralysis, as well as other nerve lesions in which deficient cell nutrition plays an important part. Damiana, by its direct action as a nerve tonic, by removing the morbid condition or stimulating the cells in inactive conditions, supplies a great want in therapeutics.

If impotency has accrued in the male from inability to secure the necessary erection to convey the seminal fluid into the female, and to produce in her the very important yet not absolutely essential orgasm for impregnation, this remedy, in the absence of organic or structural change, will almost invariably overcome the difficulty. It accomplishes all, and even more effectually, the results attained by combinations of iron, strychnia, ergot and cantharides.

In several cases of nervous exhaustion, I have found the organismal hypophosphites to give rather negative results, on account of the nerve cell being unable to imbibe its proper pabulum. In such cases I have used damiana alone with evident benefit; but the two agents together are almost magical in their effects.

I have recently used these two agents in combination with extract of malt, and the result has exceeded my fondest expectations in several cases of mal-nutrition and general cachexia. I have also noticed that the capacity of both physical and intellectual labor is increased by the use of this combination.

Recently I used damiana in a case of obstinate constipation, and found the trouble entirely removed; and this after having used a multitude of remedies. Whether the result in this case was a mere coincidence, or will again occur, I shall determine by future trials. I believe damiana can be advantageously used in all cases in which strychnia is now employed.

The preparation I have used is the fluid extract, either prepared by myself by cold repercolation, or by Dr. F. O. St. Clair. I abstain from heat in making it, as high temperature is as fatal to damiana as it is to wild cherry. May not the rise of heat in the manufacture explain the reason why so much of the fluid extracts found in the market is utterly worthless, and has brought so much reproach, to be shared by the properly prepared and valuable article?

Damiana, like ergot, isolated phosphorus compounds, dophyllin and other valuable agents, has had its good name traduced, and at it has been hurled the usual remedy of the weak ridicule; but truth, as it always will, has triumphed, and this agent is, no doubt, destined to an officinal position in our pharmacopœia.—*C. G. Polk, in Virginia Medical Monthly.*

